# Topiramate-Induced Phantosmia: A Case Report on Reversible Olfactory Hallucinations in Epilepsy Treatment

**DOI:** 10.1192/j.eurpsy.2025.743

**Published:** 2025-08-26

**Authors:** M. Peyioti, A. Karampas, V. Bountioukou, D. Varlamitis, C. Pappa, M. Plakoutsis, P. Petrikis

**Affiliations:** 1 University General Hospital of Alexandroupolis, Alexandroupolis; 2 Psychiatric Clinic, University General Hospital of Ioannina, Ioannina, Greece

## Abstract

**Introduction:**

Topiramate is a widely prescribed anticonvulsant and migraine prophylactic agent, recognized for its efficacy in treating epilepsy and preventing migraines. While its use is generally well-tolerated, it is known to cause a range of side effects. However, olfactory hallucinations, also referred to as phantosmia, represent a rare and poorly documented adverse effect of Topiramate. Phantosmia involves the perception of smells in the absence of an external odor source. This case report presents the case of a woman who developed olfactory hallucinations following the re-initiation of Topiramate for epilepsy. The hallucinations resolved completely upon discontinuation of the drug. This report highlights the need for clinicians to be aware of this potential side effect.

**Objectives:**

Topiramate is a well-established treatment for epilepsy and migraine prevention, but it can cause neurological and cognitive side effects like paresthesia, dizziness, memory issues and confusion. However, olfactory hallucinations are rare and underreported. This case report aims to contribute to the limited literature by documenting a case of reversible phantosmia related to Topiramate.

**Methods:**

A woman presented with complaints of persistent olfactory hallucinations, perceiving unpleasant odors in the absence of any external source. Her medical history was unremarkable, apart from a diagnosis of epilepsy. After other treatments had limited success, she was started on Topiramate a couple of weeks before the manifestation of the olfactory hallucinations. Weeks after starting Topiramate, the patient began experiencing olfactory hallucinations which interfered with her daily life. The patient had no prior history of olfactory disorders, nasal pathology or psychiatric conditions and her physical examination was unremarkable. Upon investigation, no structural abnormalities were found.

**Results:**

The patient’s medication history revealed that the onset of olfactory hallucinations coincided with the initiation of Topiramate.After discussing the side effect profile of the drug, it was decided to discontinue Topiramate. Within two weeks of discontinuation, the patient reported a complete cessation of the olfactory hallucinations. A follow-up evaluation showed no recurrence of the symptoms after switching to an alternative therapy. Given the temporal relationship between the onset of olfactory hallucinations and Topiramate use, alongside the resolution of symptoms upon drug discontinuation, a diagnosis of Topiramate-induced phantosmia was made.

**Conclusions:**

This case highlights a rare but significant adverse effect of Topiramate, olfactory hallucinations. Given the reversibility of this side effect upon discontinuation of the drug, physicians should be vigilant in monitoring for sensory disturbances in patients administered with Topiramate. Awareness of phantosmia as a potential adverse effect will allow for appropriate management, potentially preventing patient distress.

**Disclosure of Interest:**

None Declared

